# Avoiding Treatment Interruptions: What Role Do Australian Community Pharmacists Play?

**DOI:** 10.1371/journal.pone.0154992

**Published:** 2016-05-12

**Authors:** Salem Hasn Abukres, Kreshnik Hoti, Jeffery David Hughes

**Affiliations:** 1 School of Pharmacy, Curtin University, Perth, Western Australia, Australia; 2 Faculty of Medicine, Pharmacy Department, University of Prishtina, Prishtina, Kosovo; Azienda ospedaliero-universitaria di Perugia, ITALY

## Abstract

**Objective:**

To explore the reported practice of Australian community pharmacists when dealing with medication supply requests in absence of a valid prescription.

**Methods:**

Self-administered questionnaire was posted to 1490 randomly selected community pharmacies across all Australian states and territories. This sample was estimated to be a 20% of all Australian community pharmacies.

**Results:**

Three hundred eighty five pharmacists participated in the study (response rate achieved was 27.9% (there were 111 undelivered questionnaires). Respondents indicated that they were more likely to provide medications to regular customers without a valid prescription compared to non-regular customers (p<0.0001). However, supply was also influenced by the type of prescription and the medication requested. In the case of type of prescription (Standard, Authority or Private) this relates to the complexity/probability of obtaining a valid prescription from the prescriber at a later date (i.e. supply with an anticipated prescription). Decisions to supply and/or not supply related to medication type were more complex. For some cases, including medication with potential for abuse, the practice and/or the method of supply varied significantly according to age and gender of the pharmacist, and pharmacy location (p<0.05).

**Conclusions:**

Although being a regular customer does not guarantee a supply, results of this study reinforce the importance for patients having a regular pharmacy, where pharmacists were more likely to continue medication supply in cases of patients presenting without a valid prescription. We would suggest, more flexible legislation should be implemented to allow pharmacists to continue supplying of medication when obtaining a prescription is not practical.

## Introduction

Pharmacists in community pharmacies worldwide are often faced with customers requesting supply of prescription medication without a prescription or without a current valid prescription.[1–3] In this paper, ‘invalid prescription’ refers to an out of date prescription or a prescription without any remaining repeats. In Australia, except in the case of controlled drugs, prescriptions are valid for one year from the date of issue, or six months for controlled medications.[4] When the prescription expires, the patient must see their regular prescriber or any registered prescriber (e.g. doctor, nurse practitioner) to obtain a new prescription. However, there are situations when obtaining an appointment with the prescriber is not practical and this may result in treatment interruption.[5]

When customers run out of their prescription medicines, they may ask their regular pharmacy (or any other pharmacy) to supply their medication without a valid prescription, based on their last valid prescription. In this situation in Australia, pharmacists have the right to Not Supply (NS), or supply using: (a) Owing Prescription (OP) system, or (b) Emergency Supply (ES) system.[6] In the case of OP, the pharmacist is required to contact the prescriber to approve the OP supply, if the prescriber is not contactable, the pharmacist must not use this method. This method is funded nationally through Pharmaceutical Benefits Scheme (PBS). The PBS is a Government subsidy system for medication costs and professional fees for all Australian residents. Therefore, customers supplied with their one repeat of medication through this system are not charged beyond the usual co-payments. It requires a verbal approval (and a paper prescription within a week) by the original prescriber, and this may not be practical or possible in some situations.[5] Therefore, OP is not always available. The second method of supply (i.e. ES) does not require contact with the original prescriber. However, it only enables pharmacists to supply a limited amount of the medication and customers are charged a premium for the cost of medication (i.e. a broken pack fee) and a dispensing fee.[7] Disadvantages of the above systems have led to a new method of supply in the absence of a valid prescription, namely Continued Dispensing (CD). CD has been implemented in the majority of Australian states/territories since September 2013. CD allows the dispensing of one standard pack of the medication. However, it is currently only allowable for statin and oral contraceptive users.[5] An expanded version has been supported by its eligible users (i.e. statins and oral contraceptive users),[8] and health organizations such as the Pharmaceutical Society of Australia.[9] It is worth mentioning that at the time of conducting the present study, CD was either not implemented (i.e. time of initial survey), or had just implemented (i.e. for less than one month at the time of reminders). Therefore, this study collected information about community pharmacists’ practice before (or just shortly after) CD implementation.

Several factors may influence the method that pharmacists use to deal with medication requests without a valid prescription.[10] Firstly, the type of prescription. In Australia there are three types of prescriptions (according to what medications can be prescribed, number of repeats and funding): Standard PBS Prescriptions (Standard), PBS Authority Prescriptions (Authority) and Private Prescriptions. The PBS contains a list of medications (dispensed at a Government-subsidized price) which may be prescribed using Standard Prescriptions or Authority Prescriptions. Standard Prescriptions are the most commonly used for medications on the PBS list because they do not require a third party authorization. However, the prescriber must abide by the prescribing conditions such as the indication of use and number of repeats for individual Standard PBS items.[11] Authority Prescriptions are used to prescribe PBS listed medications which have restricted supply conditions (e.g. for a particular indication) or are prescribed in greater quantities or with more repeats than usually available through the PBS. For both prescription types, patients’ co-payments range from AU $6.10 to AU $37.70 depending on their status of concession. The rest of the medications’ costs and pharmacists’ fees are paid by the PBS.[12] Private Prescriptions are generally used to prescribe medication not listed in the PBS and/or when supply is not eligible under the PBS rules, in this case patients pay the full cost of the drug and supply.[6] Printed Standard and Authority Prescriptions are required to enable pharmacists to claim reimbursement from the PBS system, The administrative complexity to obtain a new prescription (particularly Authority Prescriptions) to cover medication supply without a valid prescription varies according to prescription type.[7] Consequently, pharmacists are likely to take this factor into their consideration in their decision to supply, and if so using what method of supply.

Secondly, customer type may also influence the method that pharmacists choose in dealing with a medication request in the absence of a valid prescription. Since pharmacies keep records of medication supply for each customer, pharmacists can review the customer’s medication dispensing history.[13] In addition, pharmacists may establish a relationship with their regular customers.[5,14] Therefore, pharmacists may provide different (and potentially more preferable) options for regular compared to non-regular customers.[2] For example supply rather than refusal to supply, or supply with OP rather than ES.

Thirdly, the type of the medication. This factor may have a positive or negative impact on pharmacist‘s decision.[2] For example, requesting an antihypertensive medication is entirely different from seeking a benzodiazepine without a valid prescription. While the motivation for requesting an antihypertensive is likely to be solely for medical reasons, the request for the benzodiazepine may not always be medically motivated.[15]

Fourthly, the frequency of request may also affect pharmacists’ decisions.[2] For example, if the customer repeatedly requested the same medication under the guise that they were unable to see their regular prescriber, this would probably reduce the likelihood that the pharmacist would provide an additional supply. However, without a fully implemented electronic health record, pharmacists may not be able to identify if a previous supply without a valid prescription was made in another pharmacy.

Finally, pharmacists’ decisions may differ according to their demographic status. Previous studies have shown that pharmacists had different practices according to their age, gender and pharmacy location.[16–18] Therefore, these factors may affected pharmacists’ decisions to supply or refuse to supply medications without a valid prescription.

This study investigated: (a) the frequency of requests by different customer types, (b) how would Australian pharmacists deal (supply or not supply and which method of supply they use) if they faced with hypothetical scenarios of customers requesting medications without a valid prescription) and (c) factors which influence their decisions.

## Methods

Australia has six states and two territories. They can be arranged according to percent of registered pharmacists as New South Wales (NSW) (31%), Queensland (QLD) (26%), Victoria (VIC) (20%), Western Australia (WA) (11%), South Australia (SA) (7%), Tasmania (TAS) (3%), Australian Capital Territory (ACT) (2%), and Northern Territory (NT) (1%). A self-administered questionnaire was sent to a randomly selected sample of community pharmacies in Australia. All pharmacies in each Australian state or territory (on the Yellow Pages website) were entered into an Excel^TM^ file, and then a random sample (20% of pharmacies) was selected using a simple random technique. A 20% sample was chosen as it allows a representative sample to be drawn from a large number of potential respondents. Therefore, 1490 questionnaires were distributed with an anticipated response rate of approximately 40% (it was anticipated to lead to approximately 600 responses, with 95% confidence level with +/-2.5 confidence interval). The randomization was done by using an electronic randomizer (http://www.randomizer.org/). The Yellow Pages website was the only readily available source to obtain postal and email addresses of Australian pharmacies. Efforts were made to obtain these addresses from licensing bodies, however, these attempts did not succeed. Sample selection depended on the number of pharmacies in each state/ territory. A total of 1490 pharmacies were selected: 464 from NSW, 378 from VIC, 326 from QLD, 135 from WA, 120 from SA, 39 from TAS, 16 from ACT and 12 from NT. The questionnaire was sent during late August 2013 by post. Whilst postal and email reminders (173 emails, i.e. those where email addresses were available) were sent out a month later (i.e. before and after implementation of the CD system in most Australian States).[19] Therefore, the questionnaire did not contain the option CD as a potential method of supply. In other words, the questionnaire explored the pharmacists’ reported practice before CD became an additional option of supply. The questionnaire did not collect information about pharmacies characteristics, such as the number of employees, the number of prescriptions dispensed or ownership of pharmacy. The postal survey was chosen because it is a cost-effective method to contact a relatively large number of pharmacies in Australia given its geographical size.[20] Participants were considered consented if they returned the questionnaire. No incentives were provided for participation.

### Questionnaire Design

Results of a literature review and experience from previous studies were used in the development of the questionnaire.[5,20] The questionnaire’s face and content validity were assessed for by piloting it with five pharmacists and eight pharmacy academic staff members working in the area of pharmacy practice at School of Pharmacy, Curtin University. After incorporation of the suggested changes, such as deletion of some questions (because they were deemed irrelevant), the final questionnaire contained 19 questions. In this manuscript, we report only five questions that cover two areas; (a) frequency of medication requests without a valid prescription by regular and non-regular customers, and (b) the reported practice by pharmacists when dealing with such requests. In addition to these areas, we also report participants’demographic information (age, gender of the participant and pharmacy location; urban (population > 100,000) or rural (population < 100,000).[21] A regular customer was defined as a customer who attended the pharmacy five times or more in the past 12 months, while the a non-regular customer was defined as a customer who: attended the pharmacy fewer than five times in the past 12 months. These definitions were obtained from the Australian Health Department website and were provided within the questionnaire.[22] The first question sought information about the frequency of customers (regular and non-regular) requesting medication(s) without a valid prescription. The second question looked at what the participants would do (NS, OP, ES or Other) when facing a medication request without a valid prescription. Therefore, they were asked to report what they would normally do when dealing with a request for each of the 19 different medication classes (See S1 Appendix). In this case the request was made by a regular customer with a stable chronic disease (as judged by the participant after consultation with the customer) and based on a previous supply with: a) Standard Prescription, b) Authority Prescription, or c) Private Prescription. Therefore, three different scenarios were used. We assumed that the pharmacist would not supply when it was not safe to do, would try to contact the original prescriber to supply using OP, or dispense a limited quantity of the requested medication using ES if the communication with the prescriber was not possible (they could also use the “Other” option to report their other actions). The third question was about the reported practice if the same customer requested the same medication (regardless the prescription and medication types) for a second time without seeing the prescriber. The fourth question was the same as the second, whilst the fifth was the same as the third, but both dealt with non-regular customers. Questions 3 and 5 were used to explore only the effect of the frequency of request and customer type on participants’practice. Therefore they were shortened to include only these factors. This has the advantage of decreasing the time needed to answer the questionnaire (which contained a total of 19 questions), without changing the intended meaning and purpose of the questions. Yet, Questions 3 and 5 provided valuable data particularly comparing participants’reported practice with regular and non-regular customers. Further details of the questionnaire are provided in the S1 Appendix (abridged version).

### Ethical Approval

This study was approved by The Human Research Ethics Committee of Curtin University (Approval number: PH-07-13).

### Data Analysis

Answers to each question were analyzed using SPSS^®^ version 22 (http://www-01.ibm.com/software/au/analytics/spss/). Responses to questions were entered into an Excel^TM^ file (See S1 Dataset), and then transferred to an SPSS^®^ data file. SPSS^®^ was used to summarize data and produce frequency tables and to describe the reported practice according to customer, prescription and medication types. Since the same participant was asked twice (i.e. first, would they supply or refuse to supply for regular customers, and second for non-regular customers) the McNemar test was used to compare the supply practices of medications between regular and non-regular customers. This test was initially utilized through the Transformed Process in the SPSS^®^ to convert responses into the dichotomous responses of Not Supply (NS) and Supply which included ES, OP and ES&OP responses. Multinomial regression was used to investigate the effect of demographic variables: age, gender, and pharmacy location description (urban or rural) on participants’ decisions to supply and what method of supply they used (i.e. ES or OP). Participants were compared according to: age for the purpose of analysis this variable was re-grouped into two groups only (the younger group i.e. ≤ 40 years vs the older group >40 years of age), gender (male vs female), and pharmacy location (urban vs rural). For all tests a p value of ≤ 0.05 was taken to indicate a statistically significant association. Multinomial regression was used because it is appropriate to model a 3-level categorical outcome variable.[23] According to Tabachnick and Fidell, “regression analysis with over approximately 150 responses are adequate to identify independent variables which exhibit a moderate effect size.”[23] It does not require a large sample and 10 cases in each variables are considered sufficient.[24] Finally, Mann-Whitney test was used to detected differences between those who responded to the initial questionnaire and those who responded to the reminder. This test was used because it is appropriate to compare two independent samples.

## Results

### Response Rate

The total questionnaires received were 385 and there were 111 undelivered questionnaires. There were 268 responses from the first mail-out and 117 from the reminder (including six via email). There were only 63 responses from the states were CD was actually implemented. No statistically significant differences (Mann-Whitney test) were detected between those who responded to the initial questionnaire and those who responded to the reminder. The overall response rate was 27.9% of delivered questionnaires. Response rates from states and territories ranged from 0% in Northern Territory to 51.4% in Tasmania.

### Demographic Data

Males were the dominant gender group of the respondents. According to age, the respondents were almost equally divided into two groups (≤40 years old and > 40 years old). The distribution of the respondents within states/territories corresponded to the number of pharmacies (hence the sample selected) in each state/territory. The participants’ primary place of work was community pharmacy (96.6%). Our demographic data is comparable to data published in a report by Health Workforce Australia 2014.[25] Further demographic details and national figures are shown in Table 1.

**Table 1:** Demographic characteristic of the participants (n = 385).

Variable	Categories	Survey Data n (%)	Australian Data^#^ n (%)
Gender	Male	210 (54.5)	8,916 (41.8)
	Female	155 (40.3)	12,415 (58.2)
	Prefer not to disclose	20 (5.2)	*NA
Age (years)	20–30	90 (23.4)	*NA
	31–40	98 (25.5)	*NA
	41–50	83 (21.6)	*NA
	51–60	83 (21.6)	*NA
	> 61	26 (1.8)	*NA
	Prefer not to disclose	5 (1.3)	*NA
State or Territory	ACT (Australian Capital Territory)	5 (1.3)	373 (1.7)
	QLD (Queensland)	92 (23.9)	4,197 (20.0)
	NSW (New South Wales)	90 (23.4)	6,584 (31.0)
	NT (Northern Territory)	0 (0)	157 (0.07)
	SA (South Australia)	31 (8.1)	1,625 (7.6)
	TAS (Tasmania)	18 (4.7)	554 (2.6)
	VIC (Victoria)	92 (23.9)	5,465 (25.6)
	WA (Western Australia)	54 (14.0)	2,367 (11.0)
	Prefer not to disclose	3 (0.8)	*NA
Pharmacy location	Urban (Metropolitan)	279 (72.5)	16,225 (76.0)
	Rural (rural, remote and other)	100 (26.0)	5,088 (24.0)
	Prefer not to disclose	6 (1.6)	*NA

# Source: Health Workforce Australia 2014,[22] according to this source the total number of pharmacists in 2012 was 21,331 working in different pharmacy settings (13,454 (63.1%) were working in community pharmacy).

*NA: not applicable

### Weekly Requests

The participants were asked to estimate the number of medication requests without a valid prescription that pharmacy received from regular and non-regular customers on a weekly basis. Four options were offered to participants to select; zero, one or two, three to four, and five or more. The most reported number of requests per week was five or more made by regular customers reported by 66.8% (n = 257) of the participants, followed by three to four times reported by 19.5% (n = 75) and one or two times reported by 8.6% (n = 33), while only 2.9% (n = 11) reported that they did not face such requests from regular customers and nine participants did not answer this question. Interestingly, one participant reported the number of requests from regular customers as 50 per week. In the case of non-regular customers, one or two requests per week was the most frequent, reported by 48.3% (n = 186) of the participants followed by five or more, reported by 21.3% (n = 82), three to four times reported by 10.1% (n = 39), and zero times was reported by 9.4% (n = 36). There were 42 participants who did not answer this question.

### The Reported Practice

The participants were asked about what they would do (*Not Supply [NS]*, *Owing Prescription [OP]*, *Emergency Supply [ES] or Other)* when dealing with hypothetical scenarios of a patient with a stable chronic disease requesting listed medications without a valid prescription, if this request was based on a previous supply with either a Standard, Authority, or Private Prescription, and was made by: (A) a regular customer or (B) an non-regular customer. The frequency of supply and the reported practice differed according to prescription, customer and medication type as outlined below.

**Fig 1:**
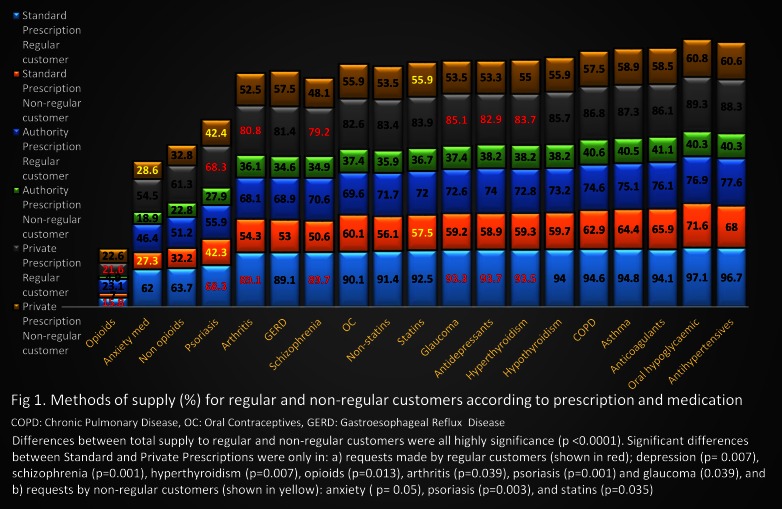
Comparison between the supply (%) for regular and non-regular customers according to prescription and medication types.

**1. Total Supply.** Fig 1 displays the overall supply according to customer, prescription and medication types. Results are summarized below.

**a. According to customer type:** Regardless of prescription or medication type, for all listed medications the likelihood of supply for regular customers was greater than for non-regular customers (Fig 1). The McNemar test was used to compare the NS and supply for each medication as dichotomous dependent variables between the two customer types as dichotomous independent variables. In all cases the difference was statistically significant (p < 0.0001), suggesting a higher rate of supply for regular customers in comparison to non-regular customers.

**b. According to prescription type:** The lowest rate of supply was for Authority Prescriptions, irrespective of customer or medication type. The highest rate of supply was associated with Standard Prescriptions (for regular customers) or Private Prescriptions (for non-regular customers), depending on medication type (Fig 1).

The McNemar test revealed statistically significant differences between the supply (or not supply), based on Authority Prescriptions and the other prescription types, for all medication types (p value < 0.05) and for each customer type. However, in a few cases there were only significant differences between Standard and Private Prescriptions (See Fig 1 for more details). Note in the case of psoriasis medication (and similar cases) for regular customers, the percentages of participants who decided to supply in both prescription types were identical (i.e. 68.3%), in these cases the significant differences arose from the differences in the numbers of participants who did not supply. For example, in the case of psoriasis medication for regular customers, there were 111 participants who selected NS in the case of Standard Prescription and 85 participants in the case of Private Prescriptions.

**c. According to medication type:** All listed medications, except opioids, were supplied by over 50% of participants regardless of prescription type, if the request for supply was made by a regular customer. If the request was made by a non-regular customer, there were more medications that would not be supplied by pharmacists in the case of Standard or Private Prescriptions (Fig 1). However, if the prescription was an Authority Prescription, the majority of the participants would not supply the listed medications.

2. The reported practice.

**Fig 2:**
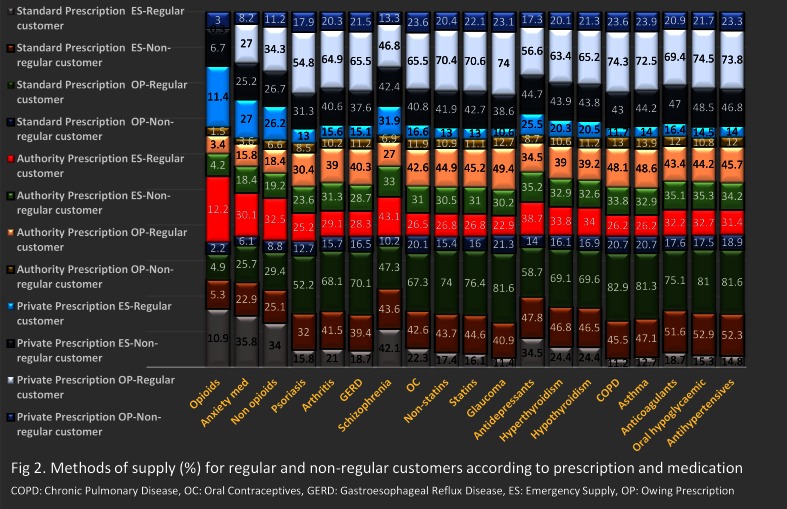
Method of supply (%) for regular and regular customers according to prescription and medication types.

**a. Regular customers:** The most reported method of medication supply was OP (Fig 2) in the case of either Standard or Private Prescriptions, except in the case of chronic pain non-opioids, antianxiety medications and opioids. In the case of Authority Prescriptions, if participants provided medications they used ES more frequently than OP; however there was a greater level of NS compared to Standard and Private Prescriptions.

**b. Non-regular customers:** In the case of non-regular customers, there was a greater incidence of NS for all prescription types, and for all types of medication. In the case of supply, the preferred method of supply was ES. In contrast to regular customers, the frequency of OP use was more for Private Prescriptions than Standard Prescriptions (See Fig 2 for more details).

### Frequency of Request

The reported practice was NS for both types of customers in cases where a second consecutive request was made before the patient obtained a new prescription. However, the likelihood of obtaining the medication was higher for regular than non-regular customers (26.3% [19.0% ES + 7.3% OP] vs 6.3% [5.5% ES + 0.8% OP], p< 0.0001).

### Effect of Some Variables on the Reported Practice

Multinomial regression analysis revealed that participants’ demographic variables (age, gender of participants and pharmacy location) had significant effects on the reported practice of how participants dealt with medication supply requests. In general and depending on the prescription and customer type, older participants were more likely than the younger participants to supply the following medications: antianxiety, non-opioids and opioids. Younger participants, however, were more likely to provide antidepressants, psoriasis medications, statins and non-statins. Participants who worked in urban areas were more likely to supply antianxiety, arthritis and Gastro-Esophageal Reflux Disease (GERD) medications than participants worked in rural areas, while male pharmacists were more likely to supply opioids than female pharmacists (Table 2).

**Table 2:** Effects of demographic variables on participants’ decisions to supply (n = 385).

Customer type	Medication	Prescription type	More likely to Supply Group	P value
Regular	Antianxiety	Standard	Older (>40 years)	0.008
	Antianxiety	Authority	Urban	0.044
	Antianxiety	Private	Older (>40 years)	0.022
	Antidepressants	Standard	Younger ≤ 40 years)	0.033
	Antidepressants	Authority	Younger (≤ 40 years)	0.005
	Antidepressants	Private	Younger (≤ 40 years)	0.023
	Non-opioids	Standard	Older (>40 years)	0.006
	Non-opioids	Authority	Older (>40 years)	0.023
	Non-opioids	Private	Older (>40 years)	0.022
	Statins	Standard	Younger (≤ 40 years)	0.007
	Statins	Authority	Younger (≤ 40 years)	0.015
	Statins	Private	Younger (≤ 40 years)	0.022
	Arthritis	Standard	Urban	0.016
	Arthritis	Authority	Urban	0.003
	Arthritis	Private	Rural	0.028
	Opioids	Authority	Older (>40 years)	0.03
	Opioids	Private	Male	0.027
	Psoriasis	Standard	Male	0.008
	Psoriasis	Private	Female	0.012
	Psoriasis	Private	Younger (≤ 40 years)	0.028
	Anticoagulants	Authority	Female	0.028
	GERD	Private	Urban	0.003
Non-regular	Statins	Standard	Younger (≤ 40 years)	0.03
	Statins	Private	Younger (≤ 40 years)	0.048
	Non-statins	Standard	Younger (≤ 40 years)	0.046

GERD: Gastro-Esophageal Reflux Disease

### Alternative Model for Medication Supply

A small number of participants (ranged from 0 to 0.08%) reported they used a hybrid model of ES and OP. In this case, the customer is provided with the minimum applicable quantity of the medication (three day’s supply or the full pack [e.g. for an inhaler medication]) and required to pay the full cost (i.e. ES) and then when the customer presents a new valid prescription they are provided with the remaining quantity and either pay or are refunded any difference between ES and PBS co-payments (i.e. OP).

## Discussion

To our knowledge, this is the first study to explore thereported practice of Australian community pharmacists when dealing with medication requests in the absence of a valid prescription. Such requests have also been reported in the international literature.[26,27] Worldwide there have been a number of changes in legislation to allow pharmacists to supply medication in urgent situations. For example, in some Canadaian Provinces, pharmacists may legally adjust the medication dose, or change the dosage form of prescribed medications to ensure treatment continauion when access to the original prescriber is not practical.[27]

Results of the current study showed that Australian community pharmacists face such requests on a weekly, if not daily, basis. Inability of customers to obtain same-day appointments was reported as a cause in both the Australian and international literature.[28,29] In a recently published Australian study, Garth et al. reported that not all patients requesting same day appointments would be able to be seen by their doctor.[28] In regards to medication requests without a valid prescription, regular customers were more likely to make such requests compared to non-regular customers (5 or more vs. 1–2 times per a week). In a previous study, requesting medication without a valid prescription was also reported by approximately one-third of patients who ran out of their medication.[5] In a recently published study from the UK, Morecroft et al. reported that requests for urgent medication supply were more likely around weekends and when other services were not available.[2] In the current study, where possible and appropriate, community pharmacists assist customers by providing an ongoing supply until they can see their doctor. They are more likely to provide ongoing supply if the medication requested is not for a drug with abuse potential, the customer is a regular and the prescription is either a Standard or Private Prescription, where obtaining a new prescription is less difficult than Authority Prescriptions. The reported practice of pharmacists dealing with medication requests in the absence of a valid prescription emphasizes the importance of customers having a regular pharmacy.[10]

Results of this study indicated that there were substantially different practices by pharmacists when dealing with hypothetical scenarios of medication requests without a valid prescription according to the customer, prescription, medication types, and frequency of the request. There were a number of factors associated with lower rates of medication supply and more usage of the ES or NS as the most reported practices. These factors included (1) non-regular customer (probably due to lack of dispensing history), (2) Authority Prescription (this seems a result of potential difficulties in obtaining a new valid prescription, which might result in breach of legal requirements and/or financial loss), and (3) medication type, in particular chronic pain non-opioids, antianxiety medications and opioids (probably because of abuse potential). Indeed, as these factors augmented, the medication supply became less frequent, resulting in more NS decisions. While the NS decision is recommended to deal with medications with abuse potential, pharmacists may find themselves in complex situations with legitimate requests for medical purposes such as patients with cancer.[15]

The ES method was the most commonly reported method of supply, in cases of the presence of one or more of the above factors, presumably because ES does not require contact with the prescriber or a future prescription. However, it is a costly method for the customer and allows only a three days’ supply. As a result of the cost,[30,31] customers may refuse to obtain their medications through ES.[15,19] Consequently, non-regular customers had a significantly lower overall rate of supply than regular customers (p < 0.0001). This is consistent with a previous study, where non-regular customers reported they ran out of their medications more frequently than regular customers.[5] The main reason related to these factors may be a potential lack of trust as reported by Hoti et al. in the pharmacist prescribing context.[32] Pharmacists may not feel confident to supply to customers for whom they do not know their medication supply history, do not trust them to accurately report their health issues and/or bring a new valid prescription back to the pharmacy, particularly in the case of Authority Prescriptions which require a third party approval.

The OP method was the most commonly reported method of supply for requests made by regular customers, based on a previous supply with a Standard or Private Prescription, and when the medication was not a chronic pain non-opioid, antianxiety medication or opioid. It is not clear whether participants used the ‘Standard OP’ procedure (i.e. supply only after the pharmacist communicated with the prescriber) or the “in advance” OP which is conducted without such communication.[5] ‘In advance’ OP is not strictly a legal method of supply; however it is used when the pharmacist is satisfied a regular customer will bring a valid prescription following their next visit to the prescriber. This is similar to ‘loan’ supply in the UK, which is used to supply medication for urgent requests without a prescription but with a future prescription anticipation. Morecroft et al. reported that avoiding additional cost to customers was one of the motivations for pharmacists’ use of this method.[2] We assume that pharmacists will firstly try to contact the prescriber and if that is not possible (thus the Standard OP is not applicable) then they may refuse to supply, use ES, or use the ‘in advance’ OP method of supply. In the case of non-regular customers, OP was less frequently used (ranged from 2.1 to 20%). This may reflect difficulty to communicate with the original prescriber of non-regular customers.

Frequency of requests also affected pharmacist’s decisions. If the same patient requested a second consecutive supply because of continued inability to see their regular prescriber the most reported practice was NS. Although participants were more flexible with their regular customers, through providing them an ES supply, compared with non-regular customers for whom the possibility to obtain a second supply was very small (p <0.0001). This may answer the question from a previous research project about “at what point does the pharmacist say, “We will no longer supply until you see the doctor?”[7] This indicates that pharmacists do not endlessly supply medication without a valid prescription and at the same time they appreciate patients’ difficulty in seeing their regular prescribers. In a previous study repeated requests was also reported as a source of distress for pharmacists, particularly if they perceived them as system abuse by patients to avoid medical review, which they have to pay for or take time off work to have done.[2]

Some demographic variables affected pharmacists’ reported practice. This effect of demographic variables is consistent with previous studies which demonstrated the association between pharmacists’ demographics and their decision making process and/ or their attitudes towards patients.[16–18] Older, males and/or pharmacists working in rural areas were more likely (than their younger, female and/or urban counterparts) to supply chronic pain non-opioids, antianxiety medications and opioids. This may have resulted from the experience of the pharmacist, where male pharmacists tended to be older than females (p < 0.05), working more on a full time basis and probably they were more likely to be a pharmacy owner.[25] Thus they may have had longer relationships with their customers and their customers’ doctors. Therefore, they may be more confident that they would obtain the anticipated prescription and/or as owners they were more able to take the financial risk of supplying medication without prescription if the anticipated prescription could not obtained. Furthermore, pharmacists working in rural areas may have had more regular customers (as result of fewer pharmacies in rural areas) and/or longer relationships (due to nature of living in rural areas) with their customers than their counterparts in urban areas.[33]

An alternative method to supply medications was also reported. As far as we know, this method has not been reported in the Australian literature before. It is a combination of both ES and OP methods. This seems to be a practical way to satisfy the customers’ needs for urgent medication supply without strictly breaking the rules, as well as avoiding any financial loss if the anticipated prescription is not obtained. Several steps have been taken by the pharmacy regulatory bodies to improve access to medication when it is impractical to obtain a renewed prescription, such CD. However, this method is restricted in terms of its frequency and its eligible medications.

Even though the sample of pharmacists was consistent with the number of pharmacies in each state/ territory the low response rate was the main limitation of this study, which is consistent with the difficulty of obtaining high response rates from healthcare professionals in general, including pharmacists.[34,35] In addition, there were a number of undelivered questionnaires which may have resulted from outdated addresses on the Yellow Pages website. Access to a more accurate mailing list and greater access to email addresses may have enhanced the response rate. The fact that there were 63 responses from states where CD had been implemented could be seen as a confounding factor. However, there were no statistically significant differences between those who responded to the first mail out and those who responded after the reminder (which was returned less than one month after CD became an available option). Moreover, CD uptake by pharmacists was reported to be very low in the first 10 months of its implementation.[19] No measures were made to identify who participated and who did not, whilst this makes a comparison between respondents and non-respondents not possible, it has the advantage of collecting more truthful data that reflects the actual behaviors of the respondents.[36] There were a large number of pairwise comparisons undertaken, which suggested the existence of a number of significant associations. Because use of the Bonferroni adjustment is not appropriate in this setting (inappropriate null hypothesis), we acknowledge that some p-values may have been less than 0.05 by chance alone (type I error). Furthermore, for statistical purposes, we compared participants according to their age as a dichotomous variable, hence decreasing error probability, however, age may not be a valid indicator of pharmacist experience. Finally, results of this study are based on reported behaviors to hypothetical scenarios rather than observation of real practice. Consequently, although the fact that the majority of participants of this study faced similar requests in their practice, minimizes the possibility of natural variance between the reports and reality, there is still a possibility that participants may deal differently with actual customers in real practice (i.e. they may have been self-reporting their ideal behaviors rather than their actual practice). Factors which contribute to the strengths of present study include: being the first to explore this area in such detailed way, the sample was selected from all Australian states and territories, and the use of hypothetical scenarios method has been proved to be effective and inexpensive tool to reflect the actual practice by healthcare professionals (doctors) with high content and face validity.[37] Moreover, anonymous reporting by study participants would eliminate “Hawthorne effect” (i.e. participants enhancing their actions under surveillance) that may occur during direct observation studies.[38] According to Evans et al.[39] case vignettes are appropriate to approximate, isolate, manipulate, and measure key aspects of the decision-making processes that individuals use in real world situations, and when “…well designed to test specific questions about judgments and decision-making, they can be highly generalizable to ‘real life’ behavior, while overcoming the ethical, practical, and scientific limitations associated with alternative methods (e.g. observation, self-report, standardized patients, archival analysis).”[39] Finally, although study generalizability may be negatively affected by the low response rate, our sample successfully reflected the national figures regarding pharmacists’ demographic information, which supports study representativeness. This study is likely to prompt further investigations of the issues raised by its findings.

Future studies should focus on exploring the use of technology in enhancing the probability of obtaining a medication supply without a valid prescription in cases where contacting the original prescriber is not practical. It should also explore ways to improve communication between healthcare professionals with the view of minimizing requests for medication in the absence of valid prescriptions.

## Conclusions

Results of the current study showed that pharmacists face requests for medications without a valid prescriptions on a weekly, if not daily basis from both regular and non-regular customers. Our results emphasize the importance of patients having a regular pharmacy to minimize medication interruption when obtaining a new prescription for a chronic medication is not practical. In advance arrangement to obtain a new prescription is highly recommended to avoid such interruptions, particularly for medications with the potential for abuse and for medication that requires authorization prior to prescribing. Moreover, models of allowing pharmacists to supply without a valid prescription, when it is safe to do, should be explored and implemented especially given that being a regular customer of a pharmacy does not guarantee supply. Future research should investigate reasons for medication requests without a valid prescription, why they are so frequent and what impact might these reasons have on pharmacists’ decisions, particularly taking into account the work by Morecroft et al. who highlighted that a perception of a “genuine mistake” can occur in different responses to a deliberate choice to try to deviate from standard practice.[2] Further, timely and cost-effective ways of communication between healthcare professionals, accessible electronic health records and/or pharmacists prescribing rights as other means of minimizing treatment interruptions resulting from the absence of valid prescriptions.

## Supporting Information

S1 Appendix Study Questionnaire. (DOCX)

S1 Dataset Raw Data. (XLS)
