# Supplementary material for: Avoiding Treatment Interruptions: What Role Do Australian Community Pharmacists Play?
Source: PLoS One. 2016 May 12;11(5):e0154992. doi: 10.1371/journal.pone.0154992 (PMC4865180; doi:10.1371/journal.pone.0154992)
Supplement: S1 Appendix — (DOCX) [file pone.0154992.s001.docx]

**Appendix 1: The Questionnaire**

**Abridged version of the questionnaire, contains only the questions used in this manuscript**

**Q1:** In the past 6 months approximately, how many times per week do you face a situation where a patient with a chronic disease requests a medication supply without a prescription because he/she is unable to obtain an appointment with the prescriber? **(**Please tick one answer for each)

|  | **Q1.A:** Requests from regular customers* | **Q1.B:** Requests from non-regular customers ^#^ |
| --- | --- | --- |
| Zero |  |  |
| One or two |  |  |
| Three to four |  |  |
| Five or more |  |  |

*Regular customers: Attends the pharmacy 5 times or more in the past 12 months,

^#^ Non-regular customers: Attended the pharmacy fewer than 5 times in the past 12 months**.**

**Q2:** If a regular customer* with a stable chronic disease requested medication without a prescription because of his/her reported inability to obtain an appointment with the prescriber what would be your practice in case of the following medications?

| Q2 | Medicine type  M= Medicine | Prescription type | | | | | | | | |
| --- | --- | --- | --- | --- | --- | --- | --- | --- | --- | --- |
|  |  | 1. Standard   prescription | | | B. Authority prescription | | | C .Private prescription | | |
|  |  | Not supply | Emergency supply | Owing prescription | Not supply | Emergency supply | Owing prescription | Not supply | Emergency  supply | Owing prescription |
| Q2.1 | Oral hypoglycemic | ⃝ | ⃝ | ⃝ | ⃝ | ⃝ | ⃝ | ⃝ | ⃝ | ⃝ |
| Q2.2 | Antihypertensive | ⃝ | ⃝ | ⃝ | ⃝ | ⃝ | ⃝ | ⃝ | ⃝ | ⃝ |
| Q2.3 | Anticoagulants | ⃝ | ⃝ | ⃝ | ⃝ | ⃝ | ⃝ | ⃝ | ⃝ | ⃝ |
| Q2.4 | Anxiety M | ⃝ | ⃝ | ⃝ | ⃝ | ⃝ | ⃝ | ⃝ | ⃝ | ⃝ |
| Q2.5 | Depression M | ⃝ | ⃝ | ⃝ | ⃝ | ⃝ | ⃝ | ⃝ | ⃝ | ⃝ |
| Q2.6 | Schizophrenia M | ⃝ | ⃝ | ⃝ | ⃝ | ⃝ | ⃝ | ⃝ | ⃝ | ⃝ |
| Q2.7 | Hypothyroidism M | ⃝ | ⃝ | ⃝ | ⃝ | ⃝ | ⃝ | ⃝ | ⃝ | ⃝ |
| Q2.8 | Hyperthyroidism M | ⃝ | ⃝ | ⃝ | ⃝ | ⃝ | ⃝ | ⃝ | ⃝ | ⃝ |
| Q2.9 | Chronic pain: opioids | ⃝ | ⃝ | ⃝ | ⃝ | ⃝ | ⃝ | ⃝ | ⃝ | ⃝ |
| Q2.10 | Chronic pain: non-opioids | ⃝ | ⃝ | ⃝ | ⃝ | ⃝ | ⃝ | ⃝ | ⃝ | ⃝ |
| Q2.11 | Asthma M | ⃝ | ⃝ | ⃝ | ⃝ | ⃝ | ⃝ | ⃝ | ⃝ | ⃝ |
| Q2.12 | Emphysema M | ⃝ | ⃝ | ⃝ | ⃝ | ⃝ | ⃝ | ⃝ | ⃝ | ⃝ |
| Q2.13 | Chronic bronchitis M | ⃝ | ⃝ | ⃝ | ⃝ | ⃝ | ⃝ | ⃝ | ⃝ | ⃝ |
| Q2.14 | Arthritis M | ⃝ | ⃝ | ⃝ | ⃝ | ⃝ | ⃝ | ⃝ | ⃝ | ⃝ |
| Q2.15 | Psoriasis/eczema M | ⃝ | ⃝ | ⃝ | ⃝ | ⃝ | ⃝ | ⃝ | ⃝ | ⃝ |
| Q2.16 | GORD M | ⃝ | ⃝ | ⃝ | ⃝ | ⃝ | ⃝ | ⃝ | ⃝ | ⃝ |
| Q2.17 | Glaucoma M | ⃝ | ⃝ | ⃝ | ⃝ | ⃝ | ⃝ | ⃝ | ⃝ | ⃝ |
| Q2.18 | Statin lipid lowering M | ⃝ | ⃝ | ⃝ | ⃝ | ⃝ | ⃝ | ⃝ | ⃝ | ⃝ |
| Q2.19 | Non-Statin lipid lowering M | ⃝ | ⃝ | ⃝ | ⃝ | ⃝ | ⃝ | ⃝ | ⃝ | ⃝ |
| Q2.20 | Oral contraceptive | ⃝ | ⃝ | ⃝ | ⃝ | ⃝ | ⃝ | ⃝ | ⃝ | ⃝ |

**Q3:** If the same regular costumer were to return for a second time asking the same medication without seeing the prescriber, because of inability to obtain an appointment what would you do?

| Not supply | ⃝ | Emergency Supply | ⃝ |
| --- | --- | --- | --- |
| Owing Prescription | ⃝ | Other: please specify: | |

**Q4.** If an non-regular customer* with a stable chronic disease requested medication without a prescription because of his/her reported inability to obtain an appointment with the prescriber, what would be your practice in case of the following medications?

| Q4 | Medicine type  M= Medicine | Prescription type | | | | | | | | |
| --- | --- | --- | --- | --- | --- | --- | --- | --- | --- | --- |
|  |  | 1. Standard   prescription | | | B. Authority prescription | | | C .Private prescription | | |
|  |  | Not supply | Emergency supply | Owing prescription | Not supply | Emergency supply | Owing prescription | Not supply | Emergency  supply | Owing prescription |
| Q4.1 | Oral hypoglycemic | ⃝ | ⃝ | ⃝ | ⃝ | ⃝ | ⃝ | ⃝ | ⃝ | ⃝ |
| Q4.2 | Antihypertensive | ⃝ | ⃝ | ⃝ | ⃝ | ⃝ | ⃝ | ⃝ | ⃝ | ⃝ |
| Q4.3 | Anticoagulants | ⃝ | ⃝ | ⃝ | ⃝ | ⃝ | ⃝ | ⃝ | ⃝ | ⃝ |
| Q4.4 | Anxiety M | ⃝ | ⃝ | ⃝ | ⃝ | ⃝ | ⃝ | ⃝ | ⃝ | ⃝ |
| Q4.5 | Depression M | ⃝ | ⃝ | ⃝ | ⃝ | ⃝ | ⃝ | ⃝ | ⃝ | ⃝ |
| Q4.6 | Schizophrenia M | ⃝ | ⃝ | ⃝ | ⃝ | ⃝ | ⃝ | ⃝ | ⃝ | ⃝ |
| Q4.7 | Hypothyroidism M | ⃝ | ⃝ | ⃝ | ⃝ | ⃝ | ⃝ | ⃝ | ⃝ | ⃝ |
| Q4.8 | Hyperthyroidism M | ⃝ | ⃝ | ⃝ | ⃝ | ⃝ | ⃝ | ⃝ | ⃝ | ⃝ |
| Q4.9 | Chronic pain: opioids | ⃝ | ⃝ | ⃝ | ⃝ | ⃝ | ⃝ | ⃝ | ⃝ | ⃝ |
| Q4.10 | Chronic pain: non-opioids | ⃝ | ⃝ | ⃝ | ⃝ | ⃝ | ⃝ | ⃝ | ⃝ | ⃝ |
| Q4.11 | Asthma M | ⃝ | ⃝ | ⃝ | ⃝ | ⃝ | ⃝ | ⃝ | ⃝ | ⃝ |
| Q4.12 | COPD | ⃝ | ⃝ | ⃝ | ⃝ | ⃝ | ⃝ | ⃝ | ⃝ | ⃝ |
| Q4.13 | Arthritis M | ⃝ | ⃝ | ⃝ | ⃝ | ⃝ | ⃝ | ⃝ | ⃝ | ⃝ |
| Q4.14 | Psoriasis | ⃝ | ⃝ | ⃝ | ⃝ | ⃝ | ⃝ | ⃝ | ⃝ | ⃝ |
| Q4.15 | GERD M | ⃝ | ⃝ | ⃝ | ⃝ | ⃝ | ⃝ | ⃝ | ⃝ | ⃝ |
| Q4.16 | Glaucoma M | ⃝ | ⃝ | ⃝ | ⃝ | ⃝ | ⃝ | ⃝ | ⃝ | ⃝ |
| Q4.17 | Statin lipid lowering M | ⃝ | ⃝ | ⃝ | ⃝ | ⃝ | ⃝ | ⃝ | ⃝ | ⃝ |
| Q4.18 | Non-Statin lipid lowering M | ⃝ | ⃝ | ⃝ | ⃝ | ⃝ | ⃝ | ⃝ | ⃝ | ⃝ |
| Q4.19 | Oral contraceptive | ⃝ | ⃝ | ⃝ | ⃝ | ⃝ | ⃝ | ⃝ | ⃝ | ⃝ |
|  |  |  |  |  |  |  |  |  |  |  |

**Q5**: If the same patient above (i.e. non-regular costumer) were to return for a second time asking the same medication without seeing the prescriber, because of inability to obtain an appointment what would you do?

| Not supply | ⃝ | Emergency Supply | ⃝ |
| --- | --- | --- | --- |
| Owing Prescription | ⃝ | Other: please specify: | |

**Part 2**: DEMOGRAPHIC INFORMATION (Please tick the appropriate answer)

**QA:** Age group: ⃝ 20-30 ⃝ 31-40 ⃝ 41-50 ⃝ 51-60 ⃝ 61 or more years ⃝ Prefer not disclose

**QB:** Gender ⃝ Male ⃝ Female ⃝ Prefer not disclose

**QC:** In which state/territory do you work?

| ACT | ⃝ | QLD | ⃝ | NSW | ⃝ | NT | ⃝ | SA | ⃝ | TAS | ⃝ | VIC | ⃝ | WA | ⃝ |
| --- | --- | --- | --- | --- | --- | --- | --- | --- | --- | --- | --- | --- | --- | --- | --- |
| Prefer to not disclose | | | | ⃝ |  | | | | | | | | | | |

**QD:** What best describes where you work?

| Capital city (State or tertiary) | ⃝ | Major regional centre | ⃝ |
| --- | --- | --- | --- |
| Country town | ⃝ | Rural | ⃝ |
| Remote | ⃝ | Other: please specify: | |
| Prefer not disclose | ⃝ |  |  |
